# Proceedings of Syracuse Hahnemannian Club

**Published:** 1888-07

**Authors:** Frederick Hooker

**Affiliations:** Secretary


### REPORT OF PROCEEDINGS OF SYRACUSE HAHNEMANNIAN CLUB.

At the regular meeting of the Syracuse Hahnemannian Club, held April 13th, there were present Drs. Hawley, Brewster, True, Robinson, Schumacker, and Hooker.

In the absence of the President the meeting came to order shortly after eight o'clock, and paragraphs 18 and 20 of the *Organon* were read.

Dr. Brewster—When I find a case where there is coldness, prostration, sore throat, heat of the hand, which becomes more intense the longer the hand is held, I say it is a case of diphtheria, whether there are patches on the throat or not.

Dr. Hawley—The allopaths make fun of us for using symptoms. How do they make a diagnosis?

Dr. True—They do not allow the word symptom. They say "subjective and objective indications."

Dr. Hawley (paragraph 20)—We cannot comprehend the dynamic action of drugs by any process of reasoning, but by actual experience. I do not think this point is sufficiently brought out. We cannot tell why potencies act by reasoning about it.

Dr. Brewster—I tell patients there is no reason in medicine.

Dr. Hawley—There is as much reason in medicine as in love.

Dr. Brewster—You cannot prove, by reasoning about it, that Ipecac produces nausea and vomiting, but experience teaches that it does.

The main fact brought out during the discussion of Picric acid was its power of producing priapism.

Dr. True reported a case of hysteria which had given him a great deal of annoyance, and which was caused by unsatisfied desire due to a weakness in the husband. He prescribed four doses of Picric acid $^{30}$ for the husband, ostensibly for toothache, with the result of completely relieving the hysteria for some time.

Adjourned to April 20th.

---

At the regular meeting, held April 20th, there were present Drs. Brewster, Hawley, True, Leggett, Robinson, and Hooker.

Dr. Seward being unable to be present, the meeting was called to order by Dr. Hawley at half-past eight o'clock.

Dr. S. L. Guild-Leggett was admitted to membership.

Paragraphs 21 and 22 of the *Organon* were read.

Dr. Leggett (paragraph 22)—The similar drug puts out the disease and leaves nature free to recover herself.

Dr. Hawley—The force (morbific) which makes a well man sick, and the force (drug) which makes a sick man well are *similar*, and repel each other so as to leave the vital force free. "Likes repel, unlikes attract."

Dr. Leggett—The simillimum is the most similar drug, in such potency that its force is just sufficient to neutralize the disease force.

Dr. Hawley—There may be many similars, but there can be only one simillimum.

Adjourned to April 27th.

---

At the regular meeting, held April 27th, there were present Drs. Brewster, Hawley, Hooker, Leggett, and Robinson.

Paragraphs 23 to 26, inclusive, of the *Organon* were read.

Dr. Brewster—Have heard that small-pox cures or eradicates all previously existing miasms.

Dr. Leggett—Dr. Kent holds that a disease (infectious) ceases to be infectious as soon as the simillimum is given.

Adjourned to May 4th.

---

At the regular meeting, held May 4th, there were present Drs. Condee, Hawley, Hooker, Robinson, Schumacker, and Sheldon.

Paragraph 27 of the *Organon* was read.

Dr. Hooker—Is it literally true that we must cover the totality of the symptoms absolutely in all cases? We often find cases where the curative remedy does not cover the totality of the symptoms absolutely.

Dr. Brewster—Remedies and symptoms which are not found in the provings.

Dr. Robinson—Do the effects of a morbid force continue after the force has ceased to act?

Dr. Hawley—How do we know that the force has ceased to act?

Dr. Hooker—Does not a drunkard feel the effects of the alcohol he has drunk after it is all eliminated from his system?

Dr. Hawley—Does not the dynamis still act?

Dr. Sheldon—Can two similar forces, one a drug force and one a disease force, co-exist in the system?

Dr. Hawley—I question whether the drug force need be stronger than the force of the disease. "Likes repel; unlikes attract;" hence the two similar forces repel each other and leave the vital force free. The drug force must be equal in energy to the disease force.

Dr. Hooker—Does not that explain the theory of aggravation? If the drug force is stronger than the disease force, does not an aggravation result?

Dr. Hawley—I think that is so.

Dr. Hooker then gave a clinical talk on Sulphur, giving the following indications:

Irritability; involuntary haste in everything; weakness of memory, especially for names. Dullness and heaviness of the head; burning heat of vertex; itching of scalp.

Gone feeling in the stomach before meals, especially at eleven A. M.; fullness of stomach after eating a little.

Rumbling and gurgling in the bowels; painless diarrhœa, driving the patient out of bed at five A. M.

Cramps in the calves and soles, especially at night; burning heat of soles at night in bed, so that the patient puts the feet outside of the bedclothes.

Raue speaks of a urinary symptom which I have not verified, but I think it peculiar, and have found it present in one case, so will give it—irresistible urging to urinate on seeing water run from a hydrant.

CASE.—Was called to see a lady who had been struck in the left eye at the outer canthus by the edge of a pear leaf, so that there was a slight laceration of the conjunctiva.

There was some inflammation of the eye, great prostration, pain and restlessness, out of all proportion to the extent of the injury. The eye symptoms seemed to call for Bell., but on "going down cellar" I found a chronic diarrhœa, driving her out of bed in the morning and without pain; burning of the soles at night, so that she would put them out of bed, and some other symptoms calling for Sulph. Sulph.$^{30}$, three or four doses every half hour, brought prompt relief, and the repetition of Sulph. a few times during an interval of several months effected a cure of the diarrhœa.

Dr. Hawley—I think the Sulphur patient not only wants the feet uncovered, but wants to press them against the wall.

Dr. Sheldon—A lady, aged sixty-five, of scrofulous diathesis, had an itch-like eruption on her hands which she suppressed with Sulphur ointment. Following the suppression she had trouble with her stomach and then an attack of facial erysipelas of the vesicular variety.

Dr. Robinson—I did not know that cold, sweaty feet indicated Sulph.

Dr. Brewster—Coming on in the evening?

Dr. Robinson—At any time.

Dr. Hawley—I regard a hot spot on the vertex the size of a half-dollar as characteristic of Sulphur.

Adjourned to May 11th.

FREDERICK HOOKER, *Secretary.*

